# Chromoblastomycosis caused by *Rhinocladiella aquaspersa*: first case report in Guatemala^☆^^☆☆^

**DOI:** 10.1016/j.abd.2019.09.014

**Published:** 2019-09-30

**Authors:** Carlos Porras-López, María Guadalupe Frías-De-León, Roberto Arenas, Erick Martínez-Herrera

**Affiliations:** a Department of Microbiology, Instituto Guatemalteco de Seguridad Social, Guatemala City, Guatemala; b Unit of Investigation, Hospital Regional de Alta Especialidad de Ixtapaluca, Ixtapaluca, Mexico; c Department of Mycology, Dr. Manuel Gea González General Hospital, Mexico City, Mexico

**Keywords:** Chromoblastomycosis, Dermatology, Mycoses

## Abstract

The authors report a case of 40-year-old male patient with a five-year history of chromoblastomycosis on his right leg. Diagnosis was performed by direct 40% KOH exam of skin scales, culture with micro- and macromorphologic analysis, and genotypic characterization (sequencing of a fragment of the ITS region and phylogenetic analysis) of the isolated fungus. *Rhinocladiella aquaspersa* was identified as the etiological agent. Initially, the treatment was with oral itraconazole 200 mg/day for one year. However, the presence of “sclerotic cells” with filaments (“Borelli spiders”) resulted in a change of medical treatment: a higher dose of itraconazole (400 mg/day) and surgery, achieving clinical and mycological cure in one year. This is the first report of chromoblastomycosis caused by *R. aquaspersa* in Guatemala.

## Introduction

Chromoblastomycosis is a skin disease and subcutaneous infection caused by the traumatic inoculation of dematiaceous fungi.^1^

Chromoblastomycosis is distributed worldwide, mainly in tropical and subtropical areas. The main etiological agents involved are *Fonsecaea pedrosoi* and *Phialophora verrucosa*, which have a worldwide distribution and are mainly present in tropical and subtropical climate areas; *Cladophialophora carrion*, which is the only species restricted to semi-arid areas with Cactaceae as foremost vegetation; and *Rhinocladiella aquaspersa*, which is considered a rare species in the Americas.^2^ In chromoblastomycosis lesions, these fungi are found as sclerotic cells. The diagnosis is established by observing the sclerotic cells in a direct examination with KOH (10–40%) or biopsy stained with hematoxylin and eosin, and is confirmed by the isolation of the fungi in culture, which makes it possible to determine the etiology. Clinical manifestations are highly variable and the current classification is based on the basic types of dermatologic lesions: nodule, tumor, verrucous, plaque, and scarring.^3^ The therapy that has shown best results is itraconazole or terbinafine combined with cryotherapy or surgery.^4^, ^5^

## Case report

**Figure 1:**
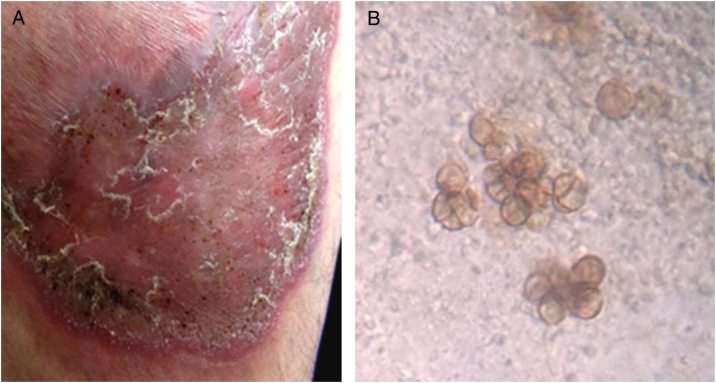
(A) Atrophic, violaceous plaque, characterized by the presence of thick scales and black dots on the borders. (B) Microscopic view of sclerotic cells in direct examination (40% KOH, 40×).

**Figure 2:**
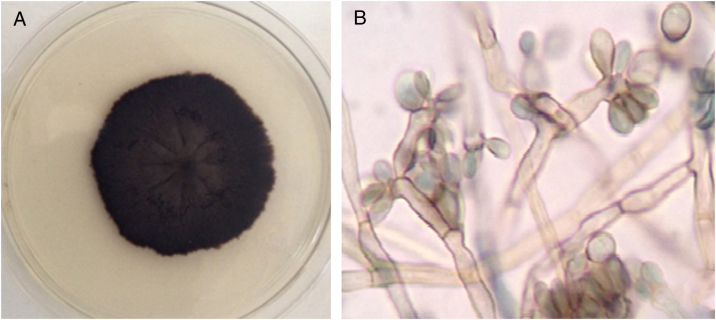
Macro-microscopic morphology compatible with *Rhinocladiella* sp. (A) Sabouraud culture demonstrates limited, hairy, velvety, and black-colored colony. (B) Septate conidiophores with multiple sympodial elliptical conidia with fine walls, showing acropleurogenous conformation.

A 40-year-old male patient, a native of Guatemala City, with no personal or family pathological background. He attended dermatological consultation due to a lesion on his right leg, characterized by a painless violaceous plaque with thick scales and black dots on the edges, of 20 cm × 35 cm in size (Fig. 1A). The patient reported the lesion appeared five years previously, when he worked as a street vendor, and he used to visit public toilets, pools, and hot springs. He remembered scratching his leg where the injury appeared during a visit to a hot spring. A punch biopsy was made; direct microscopy for mycological examination of skin fragments soaked in 40% KOH revealed the presence of sclerotic cells (Fig. 1B). Furthermore, a scraping of the plaque was performed, and the scales obtained were inoculated in Sabouraud agar at 28 °C, for four weeks. The growth of a black-colored, limited, downy, velvet colony was observed. The isolated fungus was named as 450GT. The micromorphological analysis of the isolate revealed structures compatible with *Rhinocladiella* spp.: septate, ellipsoid conidiophores with multiple sympodial conidia with slim walls, and with acropleurogenous conformation (Fig. 2). These findings confirmed the diagnosis of chromoblastomycosis, so the treatment with itraconazole (200 mg/day/six months) was started.

To determine the fungus species isolated, a fragment of 632 bp was amplified with the primers ITS1 (5′-TCCGTAGGTGAACCTGCGG-3′) and ITS4 (5′-TCCTCCGCTTATTGATATGC-3)^6^; the amplicon was sequenced in both sense strands (Macrogen – United States). The sequence was deposited in the GenBank database (*Rhinocladiella aquaspersa* Access No. MG996793).

**Figure 3:**
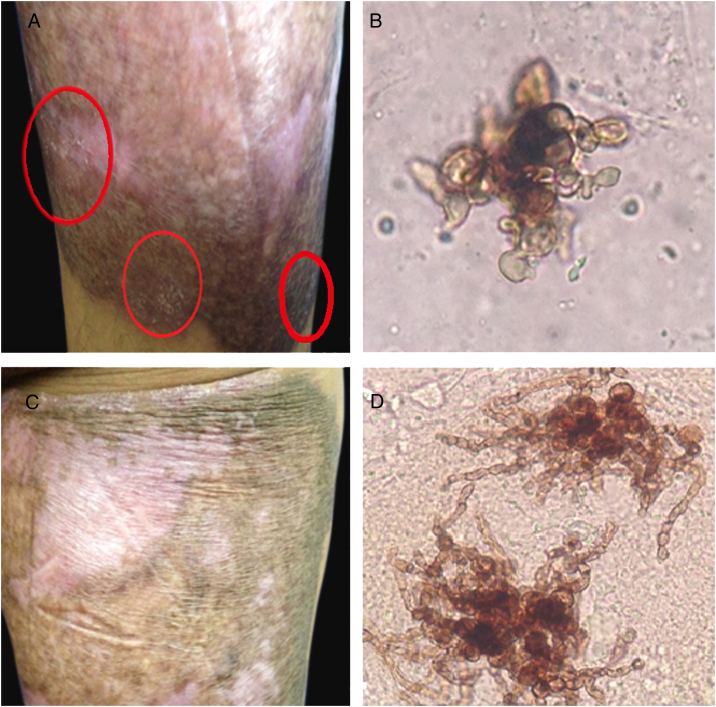
Six months of therapy with itraconazole. (A) Plaque border with fine scaling, and black dots. (B) Sclerotic cells with emission of short filaments or “Borelli spiders”. One year after treatment with itraconazole. (C) Scaling on the superior border of the plaque. (D) Sclerotic cells with emission of long filaments or “Borelli spiders”.

When the sixth month of therapy with itraconazole ended, a small area at the edges that showed fine scaling and black dots was observed. The presence of the fungus was confirmed by the observation of sclerotic cells with emission of short filaments (Fig. 3A and B) known as “Borelli spiders.”^7^ Hence, the treatment was continued for six more months. One year after the first consultation and having concluded the treatment with itraconazole (200 mg/day), the patient presented a lesion that suggested active disease. Direct examination of the lesion again showed the sclerotic cells with filaments, but longer (Fig. 3C and D). Therefore, surgical excision was carried out and treatment with itraconazole (400 mg/day) was continued for one more year, achieving mycological and clinical cure.

## Discussion

The present case report corresponds to superficial chromoblastomycosis (in plaque), whose diagnosis was suspected by the presence of black dots on the edges of the plaque and thick scales. This was later confirmed by the presence of sclerotic cells, and the isolation of dematiaceous fungus. In this case, the presence of sclerotic cells with filaments known as “Borelli spiders” stands out, which is attributed to the fact that at the level of the stratum corneum, sclerotic cells practically “germinate,” forming hyphae.^8^ Likewise, the presence of a substantial number of scales could serve as a culture medium that helps the development of hyphae or filaments derived from sclerotic cells, but without the growth of fructification forms, which occurs *in vitro*.

Therefore, it is important to perform the diagnosis based on the identification of the etiological agent at the species level to provide the best treatment and for better prognosis of the disease. In this study, in addition to the phenotypic characterization, a phylogenetic analysis was performed, using a sequence of the ITS region. This analysis showed a clear difference between the etiological agents of chromoblastomycosis and revealed *R. aquaspersa* as the cause of the mycosis in the patient. Therefore, the ITS region is a useful tool for the accurate diagnosis of chromoblastomycosis.

Based on the identification of the pathogen, itraconazole (200 mg/day) was used. During the follow-up of the patient, it was observed that a therapy of six months was ineffective, since sclerotic cells with short filaments were observed, which forced treatment to continue with itraconazole for six more months; however, after one year of treatment, “Borelli spiders”^7^ could still be seen. Therefore, it was necessary to indicate surgical treatment and a higher dose of itraconazole (400 mg/day), for one more year, to achieve mycological and clinical cure.^9^

In the present case, the association between the mycosis and the patient's occupation or his residence (Guatemala City) was not clear. The patient reported having suffered a skin abrasion on his affected leg during a bath in a hot spring, so this was the likely route of infection, since the fungus can grow in hostile habitats.^3^

The treatment of chromoblastomycosis is a challenge. In this case, monotherapy was not successful and surgery was required since the presence of filaments makes cure more difficult.^10^

This is the first case of chromoblastomycosis by *R. aquaspersa* reported in Guatemala.

## Financial support

None declared.

## Author's contribution

Carlos Porras-López: Elaboration and writing of the manuscript; obtaining, analyzing and interpreting the data; effective participation in research orientation; intellectual participation in propaedeutic and/or therapeutic conduct of the cases studied.

María Guadalupe Frías-De-León: Elaboration and writing of the manuscript; obtaining, analyzing and interpreting the data; effective participation in research orientation; critical review of the literature.

Roberto Arenas: Approval of the final version of the manuscript; effective participation in research orientation; intellectual participation in propaedeutic and/or therapeutic conduct of the cases studied; critical review of the manuscript.

Erick Martínez-Herrera: Approval of the final version of the manuscript; effective participation in research orientation; intellectual participation in propaedeutic and/or therapeutic conduct of the cases studied; critical review of the literature; critical review of the manuscript.

## Conflicts of interest

None declared.
